# Social media heterogeneity and preventive behaviours during the COVID-19 outbreak: a survey on online shopping

**DOI:** 10.1186/s12889-024-18253-y

**Published:** 2024-04-29

**Authors:** Hu Xue, Xiaoning Li, Yuye Yang, Ying Liu, Xianhui Geng

**Affiliations:** 1 College of Economics and Management, Nanjing Agricultural University, Nanjing, 210095 China; 2 School of Basic Medicine, Naval Medical University, Shanghai, 200433 China

**Keywords:** COVID-19, Preventive behaviour, Online shopping, Social media, Information source, Information content

## Abstract

**Background:**

Residents’ adoption of preventive behaviours proved beneficial in preventing the large-scale transmission of the virus during the early stages of the COVID-19 outbreak. It is critical to investigate how social media triggers residents' preventive behaviour decisions during the COVID-19 outbreak.

**Methods:**

This paper selected online shopping as a specific preventive behaviour for empirical investigation. An online cross-sectional survey was conducted through the Sojump website from 1 to 15 March 2020, and a total of 1,289 valid questionnaires were collected from China. This paper uses multiple regression analysis to investigate the heterogeneous impacts of different information sources on residents' online shopping willingness and online shopping behaviour and the heterogeneous impacts of different information content in social media on the transformation of residents' online shopping willingness and online shopping behaviour.

**Results:**

The findings indicate that both official-media and self-media positively promote residents' online shopping willingness and behaviour, with official-media having a stronger promotional effect than self-media. Furthermore, official-media and self-media can collaboratively promote residents' online shopping willingness and online shopping behaviour. The ease-of-use and usefulness of information significantly promoted the transformation of residents' online shopping willingness.

**Conclusions:**

This study analyses the heterogeneous impacts of social media on residents' preventive behaviours from the perspectives of information source differentiation and information content differentiation, which enriches related studies and provides feasible paths for promoting residents' preventive behaviours.

**Supplementary Information:**

The online version contains supplementary material available at 10.1186/s12889-024-18253-y.

## Background

In late 2019, the large-scale outbreak of coronavirus disease 2019 (COVID-19), became the most serious abrupt public health event in China and worldwide since the SARS-CoV-2 infection. Residents' adoption of preventive behaviours was the most effective emergency reaction during the early stages of the outbreak, which is critical for preventing large-scale transmission of the virus [1]. Residents' self-control weakened early during the COVID-19 outbreak as a result of their inability to understand the virus's origins and predict the spread of the disease. Risk information is thought to be the initial factor influencing residents' behaviour, triggering their cognitive processes [2]. Therefore, it is urgent to compensate for the self-control gap by obtaining risk information about the COVID-19 outbreak. Social media is the main channel through which residents obtain risk information, especially for emerging diseases, as most residents lack direct experience [3]. Social media is currently considered to be an important component of resolving public crises [4].

Previous research on the effectiveness of risk communication has generally lacked attention on the diversity of information sources on social media. With the rapid development of information technology, social media has become more diversified. On the one hand, residents open accounts on social media to share their opinions, and self-media play the role of civil opinion advocates [5]. On the other hand, traditional media and government organizations have created accounts on social media, such as microblogs and WeChat; thus, social media has gradually become the distribution channel for official information [6]. On this basis, user identities are distinguished on social media, with government and official (mainstream) accounts serving as official-media and individual accounts serving as self-media. As social media outlets are significantly different in terms of their correctness, accuracy, and diversity of information, residents have different perceptions of information from different sources, which affects their preventive behaviour decisions [7, 8]. A realistic question remains to be answered. Are there differences in the influence of different information sources in social media (official-media and self-media) on residents' preventive behaviours? How well do official-media and self-media together influence residents' preventive behaviours when they coexist on social media?

In the context of preventive behaviours (such as wearing medical masks, frequent hand washing, social distancing, etc.), it is evident that a great percentage of residents intend to act in safe ways, but only some of these residents will act on this intent [9]. Empirical support for the relationship between preventive behaviour willingness and actual preventive behaviour is weak [10], indicating that there may be other factors that explain why some residents were willing to adopt preventive behaviours but did not. The Technology Acceptance Model (TAM) assumes that, having formed an intention to act, people will act on those intentions [11]. In reality, however, the adoption of preventive behaviours may not be a direct or immediate consequence of residents' willingness, as it is subject to a wide range of other constraints. This gap between willingness and behaviour could be attributed to differences in perceptions [9]. According to the TAM, perceived ease-of-use and perceived usefulness can significantly enhance behavioural control in adopting preventive behaviours, which in turn can facilitate the transformation of preventive behaviour willingness to actual preventive behaviour [12]. Therefore, does social media delivery of relevant information to residents facilitate the transformation of preventive behaviour willingness to actual preventive behaviour?

This study selected online shopping as a specific preventive behaviour for empirical investigation. The reason is that online shopping can interrupt the transmission chain of the COVID-19 virus due to zero-contact characteristics and thus help the government control the massive spread of the COVID-19 virus [13]. An online survey by Chang and Meyerhoefer [14] indicated that residents actively adopted online shopping during the COVID-19 outbreak. Moreover, according to data from the National Bureau of Statistics of China [15], from January to April 2020, the total retail sales of resident goods amounted to $1483.6 billion, down 16.2 percent in nominal terms, but the national online retail sales of physical goods amounted to $357.8 billion, up 8.6 percent in nominal terms. A review of the above evidence indicates that the COVID-19 outbreak has stimulated residents to shop online.

This paper aims to evaluate the impact of social media on residents' preventive behaviours during the COVID-19 outbreak. As a result, two research questions were formulated: (1) Are there differences in the influence of different information sources on social media (official-media and self-media) on residents' preventive behaviours? How well do official-media and self-media together influence residents' preventive behaviours when they coexist on social media? (2) Does social media delivery of relevant information to residents facilitate the transformation of preventive behaviour willingness to actual preventive behaviour?

## Theory and hypotheses

### Technology acceptance model

**Fig. 1:**
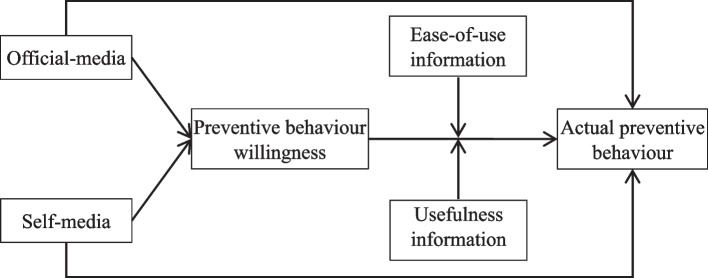
Research framework

Based on the theory of reasoned action (TRA) and the theory of planned behaviour (TPB), Davis (1989) [16] proposed the TAM, which was initially used to explain the acceptance of a technology innovation across a variety of circumstances. Because of its simplicity, this TAM is one of the most efficient models for predicting technology adoption [17]. If online shopping is considered a protective technology against COVID-19 viral infections, the TAM could offer a clear-cut and efficient method for examining the acceptance of this behaviour. Empirical research has shown the validity of the TAM. Numerous studies have used the TAM as their framework for predicting residents' online shopping willingness [18, 19]. However, TAM has several intrinsic limitations. Perceptions of the TAM were reported to be critical predictors of online shopping adoption. The current literature has widely applied risk perception in the research on the formation mechanism of residents' online shopping and indicates that risk perception is the main factor influencing residents' online shopping [20, 21]. However, risk perception is not the initial factor influencing residents' online shopping. To formulate effective risk countermeasures that can guide residents to adopt online shopping to prevent them from being infected by the virus, we must identify initial factors that influence residents' online shopping. There is a gap between residents' online shopping willingness and online shopping behaviour. Nevertheless, the linkage between intention and usage is frequently overlooked in TAM-based research studies since they tend to adopt intentions rather than actual use as outcome variables [22]. Consequently, although the TAM has generally been a rigorously tested model for predicting user acceptance of online shopping, some have raised the need for the model to be extended and incorporated with further constructs to enhance its explanation and prediction of online shopping adoption [23]. An adapted TAM framework (refer to Fig. 1) was developed to understand how social media triggers residents' preventive behaviour decisions during the COVID-19 outbreak.

#### Different information sources in social media

Previous studies have generally agreed that the actions of residents' responses to risk usually follow the pattern of “information search—perception formation—taking action” [24]. Appropriate information delivery not only encourages residents to engage in preventive behaviours but also helps them engage in appropriate preventive behaviours. Zhang [25] found that social media plays an important role in the dissemination of risk information and the shaping of public risk perception. During the COVID-19 outbreak, residents actively acquired risk information on social media, and this information encouraged them to adopt preventive measures [26]. Exposure to risk information through social media may be intentional or accidental, i.e., active or passive [27]. By acquiring risk information, residents could improve their understanding of COVID-19 [28] and reduce uncertainties, which could help them take preventative action against the COVID-19 outbreak [29, 30].

Residents have different perceptions of information from different communication channels, which affects their risk perception and online shopping decision-making [31]. Consumers who are more inclined to accept risk information with high credibility are more likely to be accepted by consumers [32]. In comparison, risk information with low credibility is difficult to obtain, and consumers have to more systematically analyse this risk information [33]. Extremely credible (or powerful) information may lead consumers to immediately or unquestionably shop online, even if there is no explanation for why protective behaviours are necessary [34]. Previous studies have indicated that official-media is more credible than self-media [10].

##### Hypothesis 1

Compared to self-media, official-media are more likely to promote residents' protective behaviours.

At the early stages of the COVID-19 outbreak, there was a relative lag when official-media provided risk information [35]. If officials’ media response to risks is insufficient, residents' negative emotions increase, increasing their reluctance to adopt preventive behaviours [36]. In contrast, self-media, by virtue of its proximity, low professional access threshold, and instant, decentralized, dynamic, and fast dissemination characteristics, successfully broke through the qualitative mode of traditional media production, dissemination, and control, becoming the main channel for the public to obtain and disseminate epidemic information on COVID-19 [34]. However, it was difficult to guarantee and control the accuracy and authenticity of circulating information during this process because unverified information spreads at an uncontrollable rate [37]. Additionally, residents were influenced by a large amount of mixed information, making it challenging for them to distinguish between scientific evidence and unreliable information [38]. Therefore, self-media can compensate for official-media's slow dissemination, whereas official-media can compensate for self-media's lack of credibility.

##### Hypothesis 2

The official-media and self-media can collaboratively promote residents' protective behaviours.

#### Different information contents in social media

Previous studies have implicitly assumed that preventive behaviour willingness and actual preventive behaviour are consistent. However, preventive behaviour willingness and actual preventive behaviour are not equivalent in terms of decision-making processes [8]. For residents, whether they can be persuaded to adopt preventive behaviour depends on the degree of effort they are willing to spend on information processing [39]. According to the Technology Acceptance Model (TAM), perceived ease-of-use and perceived usefulness can significantly enhance behavioural control in adopting preventive behaviours, which in turn can facilitate the transformation of preventive behaviour willingness to actual preventive behaviour [12]. Therefore, social media delivery of relevant information to residents facilitates the transformation of preventive behaviour willingness to actual preventive behaviour.

Perceived ease-of-use is defined as the degree to which an individual believes that using a particular technology will be free of effort. Rodrigues et al. [40] found that the success of an online service significantly depends on its ease-of-use within the TAM framework. During the COVID-19 pandemic, scholars reported similar results. For example, Wu and Song [19] reported that when residents find a service easy to use and help them protect themselves during a pandemic, they will positively adopt it. In the future, social media that disseminates ease-of-use information about preventive behaviour might become prevalent due to the rising level of digital literacy among residents [41]. Soh et al. [42] explored this topic in the nonpandemic context, and their research identified a positive relationship between ease-of-use and residents’ perceptions, acceptance and willingness towards online shopping.

Perceived usefulness is defined as the degree to which an individual believes that using a particular technology will enhance his or her performance. In other words, residents are more likely to adopt preventive behaviour when they believe it will help them attain gains in some aspect. In this study, perceived usefulness is interpreted as residents’ perception that online shopping has its own advantages as a means of personal protection during the COVID-19 outbreak; i.e., residents tend to shop online when they feel that online shopping provides indisputable protective effects. Previous research has shown that residents are more likely to use and accept online shopping if they perceive its usefulness and beneficial effects [43]. Within the TAM framework, residents’ attitudes towards online shopping are more favourable when the technology is useful and social distancing helps to protect residents from infection [44]. In addition, residents have limited access to physical stores because of the embargo, and online shopping can address residents' daily shopping needs [45].

##### Hypothesis 3

Social media provides ease-of-use information about preventive behaviour and can promote the transformation of preventive behaviour willingness to actual preventive behaviour.

##### Hypothesis 4

Social media providing usefulness information about preventive behaviour can promote the transformation of preventive behaviour willingness to actual preventive behaviour.

## Methods

### Data sources

Given that all residents were required to be home-isolated during the COVID-19 outbreak, the questionnaire survey was conducted online, as it was the only available avenue for data collection at the time. An online cross-sectional survey was conducted through the Sojump website (https://www.wjx.com) from 1 to 15 March 2020, and a total of 1,289 valid questionnaires were collected from China. The Sojump website is one of the leading online survey platforms in China [32]. It has more than three million active sample resources all over China with diverse demographic backgrounds. Each valid participant of this study was rewarded with 7 RMB (approximately one US$) for their effort. The reward-based approach could help to increase the response rate and reduce the occurrence of invalid samples [46]. Furthermore, although our study was carried out only in China, our sample was very heterogeneous, covering 31 provinces in China, and our sample size was large enough to achieve valid results for all groups [47]. The study was open to Chinese adults who were at least 18 years old. The poll was optional and anonymous. The study was was granted an exemption from requiring ethics approval by the Institutional Review Board (IRB) of Nanjing Agricultural University. Informed consent was obtained from all participants through online responses before the start of the survey.

### Measurement

#### Preventive behaviour

This study selected online shopping as a specific preventive behaviour for empirical evaluation. We asked the respondents to report their online shopping willingness and online shopping behaviour. The measure of online shopping willingness was assessed with the following question: “What percentage of daily necessities are you willing to shop online during the COVID-19 outbreak?”. Online shopping willingness was measured on a scale ranging from 0 to 100. Online shopping behaviour was measured by the following question: “What percentage of your daily necessities did you shop online during the COVID-19 outbreak?”. Online shopping behaviour was measured on a scale ranging from 0 to 100.

#### Information sources in social media: official-media and self-media

As social media outlets are significantly different in terms of their correctness, accuracy, and diversity of information, residents have different perceptions of information from different sources, which affects their preventive behaviour decisions [7, 8]. User identities are distinguished on social media, with government and official (mainstream) accounts as official-media and individual accounts as self-media. Therefore, information sources in social media comprise two dimensions: official-media and self-media. The measure of official-media was assessed with the following question: “How much information about the COVID-19 outbreak do you get from official-media during the COVID-19 outbreak?”, which was measured on a 5-point Likert-type scale ranging from 1 (not at all) to 5 (very great extent). Self-media was assessed with the following question: “How much information about the COVID-19 outbreak do you get from self-media during the COVID-19 outbreak?”, which was measured on a 5-point Likert-type scale ranging from 1 (not at all) to 5 (very great extent).

#### Information content in social media: usefulness information and ease-of-use information

Perceived ease-of-use and perceived usefulness can significantly enhance behavioural control in adopting preventive behaviours, which in turn can facilitate the transformation of preventive behaviour willingness to actual preventive behaviour [12]. Social media delivery of relevant information to residents facilitates the transformation of preventive behaviour willingness to actual preventive behaviour. Therefore, the information content in social media comprises two dimensions: usefulness information and ease-of-use information. The usefulness information was assessed with the following question: “How much usefulness information about online shopping did you get during the COVID-19 outbreak?”, which was measured on a 5-point Likert-type scale ranging from 1 (not at all) to 5 (very great extent). The ease-of-use information was assessed with the following question: “How much ease-of-use information about online shopping did you get during the COVID-19 outbreak?”, which was measured on a 5-point Likert-type scale ranging from 1 (not at all) to 5 (very great extent).

## Results

### Descriptive statistics

Table 1 reports the demographic descriptions of the respondents. Approximately 54.77% of the respondents were female. Approximately 29.87% of the respondents were aged between 26 and 30 years, and approximately 42.05% were aged between 31 and 40 years. In terms of education level, approximately 75.64% of the respondents had a university degree (undergraduate or graduate). In terms of monthly household income, approximately 16.21% of respondents had incomes between 9001 Chinese yuan and 11,000 Chinese yuan, while approximately 20.02% had incomes between 11,001 Chinese yuan and 15,000 Chinese yuan. Approximately 69.98% of the respondents lived with minors, and approximately 55.78% of the respondents lived with elderly people. In terms of location of residence, approximately 64% of the respondents lived in cities, approximately 23.2% of respondents lived in towns, and approximately 12.8% of respondents lived in villages.

**Table 1:** Demographic profile of respondents (*N* = 1289)

Variable	Measure	Frequency	Percentage(%)
Gender	Male	583	45.23
	Female	706	54.77
Age	18–25	175	13.58
	26–30	385	29.87
	31–40	542	42.05
	41–50	138	10.71
	More than 50	49	3.8
Education	Less than high school	13	1.01
	High school	79	6.13
	Vocational school	222	17.22
	College graduate	867	67.26
	Masters degrees or PhD	108	8.38
Mouthly income, Chinese yuan (US $)	≤ 5001 (≤ 700)	95	7.37
	5001–7000 (700–980)	194	15.05
	7001–9000 (980–1260)	178	13.81
	9001–11000 (1260–1540)	209	16.21
	11,001–15000 (1540–2100)	258	20.02
	15,001–20000 (2100–2800)	190	14.74
	≥ 20,000 (≥ 2800)	165	12.8
Are there older people living together?	Yes	719	55.78
	No	570	44.22
Are there children living together?	Yes	902	69.98
	No	387	30.02
Location	City	825	64
	Town	299	23.2
	Village	165	12.8

### Descriptive analysis of residents' online shopping willingness and behaviour

**Fig. 2:**
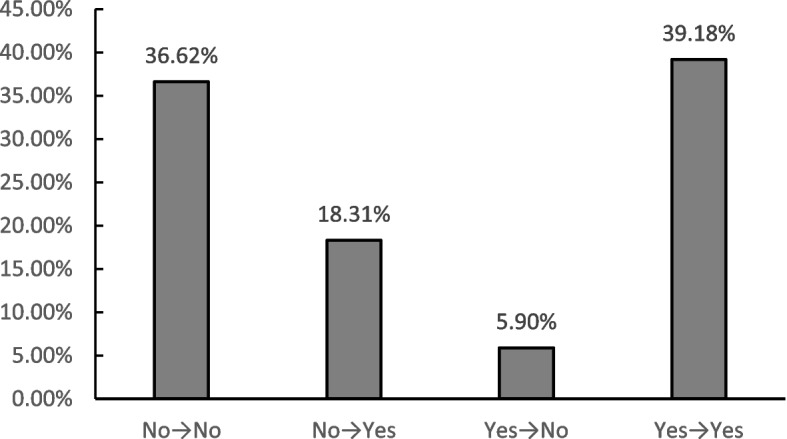
Evolution of residents' online shopping behaviour before and after the COVID-19 outbreak (*N* = 1289)

To examine the changes in residents' online shopping, participants were asked about their online shopping before the COVID-19 outbreak and during the COVID-19 outbreak. Figure 2 shows the changes in residents' online shopping before the COVID-19 outbreak and during the COVID-19 outbreak. According to the findings in Fig. 2, the percentage of residents who reported online shopping increased after the COVID-19 outbreak, increasing from 45.07% to 57.49%, which is consistent with the empirical results of Chang et al. [14] and Hao et al. [48]. As shown in Fig. 2, 18.31% of the respondents in the "No → Yes" group had no online shopping experience before the COVID-19 outbreak but adopted online shopping during the COVID-19 outbreak. Residents rapidly adopted online shopping as a way to protect themselves from the virus during the COVID-19 outbreak [49] because online shopping refers to residents’ purchase behaviours that minimize face-to-face contact with other people (e.g., employees, shoppers) during the course of shopping [13], which significantly reduces the probability of transmission of the virus from person to person. Moreover, some residents stopped shopping online. The reason is that during the COVID-19 outbreak, the external situation of residents' online shopping was poor, and they were located in areas where online shopping channels were inconvenient and inaccessible.

**Fig. 3:**
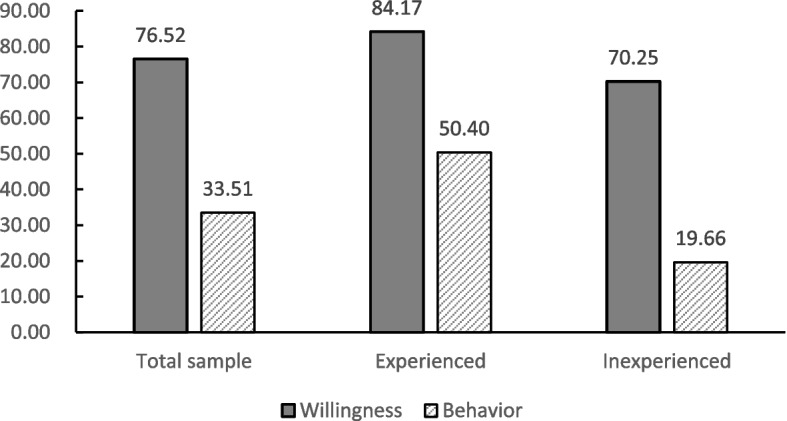
Status of residents' online shopping willingness and behaviour during the COVID-19 outbreak (*N* = 1289)

Figure 3 shows the status of residents' online shopping willingness and online shopping behaviour during the COVID-19 outbreak. The results in Fig. 3 show that there is a deviation between residents' online shopping willingness and online shopping behaviour. The reason is that the actual implementation of online shopping not only depends on residents' online shopping willingness but also on the conditions that may prevent them from adopting online shopping in the social environment [50]. Compared to the sample of residents with online shopping experience, the sample of residents without online shopping experience had lower willingness and behaviour to shop online, and the difference between online shopping willingness and behaviour was greater.

### The heterogeneous effects of different information sources on social media coverage on residents' online shopping willingness and online shopping behaviour

cMultiple regression analysis was used to test the heterogeneous effects of different information sources on residents' online shopping willingness and online shopping behaviour (Supplementary Model 1–4). According to Model 1 and Model 3 of Table 2, the results showed that the influence of official-media (*β* = 2.2306, *p* < 0.01; *β* = 6.3691, *p* < 0.01) and self-media (*β* = 1.6485, *p* < 0.01; *β* = 3.9803, *p* < 0.01) are positively and significantly related to residents' online shopping willingness and online shopping behaviour. That is, the more risk information about COVID-19 is provided by official-media and self-media, the greater the willingness of residents to shop online and online shopping behaviour. In addition, the estimated coefficient of the official-media is larger than the estimated coefficient of the self-media, which indicates that the promotion effect of risk information provided by the official-media is greater for residents' online shopping willingness and online shopping behaviour than for that provided by self-media, thus supporting Hypothesis 1. According to Model 2 and Model 4 of Table 2, the interaction term between the official-media and self-media (*β* = 1.3831, *p* < 0.01; *β* = 3.8668, *p* < 0.01) has a positive and significant effect on residents' online shopping willingness and online shopping behaviour, which indicates that the former can collaboratively promote residents' online shopping willingness and online shopping behaviour, thus supporting Hypothesis 2.

**Table 2:** The heterogeneous effects of different information sources

Dependent variable	Online shopping willingness		Online shopping behaviour	
	Model 1	Model2	Model3	Model4
*Official-media*	2.2306^a^ (0.4709)	2.5051^a^ (0.4753)	6.3691^a^ (0.7253)	7.1364^a^ (0.7239)
*Self-media*	1.6485^a^ (0.3614)	1.8952^a^ (0.3666)	3.9803^a^ (0.5566)	4.6701^a^ (0.5584)
*Official-media × Self-media*		1.3831^a^ (0.3943)		3.8668^a^ (0.6006)
*Gender*	0.1521 (1.1106)	0.3426 (1.1070)	3.6752^b^ (1.7106)	4.2078^b^ (1.6862)
*Age*	0.4976 (0.5782)	0.4666 (0.5757)	1.5698^c^ (0.8905)	1.4832^c^ (0.8769)
*Education*	1.2807 (0.8083)	1.3536^c^ (0.8050)	2.4445^b^ (1.2450)	2.6484^b^ (1.2261)
*Mouthly income*	1.3286^a^ (0.3344)	1.2995^a^ (0.3330)	3.0349^a^ (0.5150)	2.9533^a^ (0.5072)
*Are there older people living together?*	-1.7782 (1.1527)	-1.8405 (1.1478)	-0.0625 (1.7755)	-0.2367 (1.7483)
*Are there children living together?*	-1.4721 (1.2599)	-1.2943 (1.2554)	-3.1049 (1.9406)	-2.6077 (1.9122)
Location	-2.3791^a^ (0.5611)	-2.3573^a^ (0.5586)	-4.7629^a^ (0.8642)	-4.7020^a^ (0.8509)
Constant	59.6061^a^ (5.2480)	56.8846^a^ (5.2823)	-21.0316^a^ (8.0835)	-28.6404^a^ (8.0458)
Adjust *R*-squared	0.0887	0.1946	0.0966	0.2192
Observations	1289	1289	1289	1289

^a, b, c^ represents significant at 1%, 5% and 10% levels respectively, with standard errors in brackets

### The heterogeneous impact of different information content in social media on the transformation of residents' online shopping willingness and online shopping behaviour

This study tests the heterogeneous impact of different information content on social media on the transformation of residents' online shopping willingness and online shopping behaviour (Supplementary Model 5–6) through multiple regression analysis. According to Model 6 of Table 3 below, online shopping information on usefulness and ease-of-use significantly promoted the transformation of residents' online shopping willingness and online shopping behaviour (*β* = 0.1748, *p* < 0.01 and *β* = 0.0697, *p* < 0.05, respectively), which indicated that the more online shopping information on usefulness and ease-of-use was obtained, the greater the transformation of residents' online shopping willingness and online shopping behaviour was, thus supporting Hypothesis 3 and Hypothesis 4.

**Table 3:** The heterogeneous impact of different information content

Dependent variable	Online shopping behaviour	
	Model 5	Model 6
*Online shopping willingness*	0.7493^***^ (0.0423)	0.7848^***^ (0.0424)
*Usefulness information*	3.0110^***^ (0.9174)	4.2361^***^ (0.9517)
*Ease-of-use information*	1.7739^***^ (0.6444)	1.2903^**^ (0.6455)
*Online shopping willingness × Usefulness information*		0.1748^***^ (0.0333)
*Online shopping willingness × Ease-of-use information*		0.0697^**^ (0.0295)
*Official-media*	4.3654^***^ (0.6186)	4.0699^***^ (0.6169)
*Self-media*	2.7194^***^ (0.4751)	2.7572^***^ (0.4699)
*Gender*	3.6057^**^ (1.4434)	3.7410^***^ (1.4280)
*Age*	1.0594 (0.7519)	0.9259 (0.7441)
*Education*	1.2878 (1.0516)	1.2157 (1.0421)
*Mouthly income*	1.7812^***^ (0.4396)	1.8250^***^ (0.4352)
*Are there older people living together?*	1.2492 (1.5009)	1.2542 (1.4857)
*Are there children living together?*	-1.9770 (1.6398)	-1.6916 (1.6226)
*Location*	-2.9909^***^ (0.7351)	-2.7208^***^ (0.7289)
*Constant*	-79.3694^***^ (7.5237)	-87.0045^***^ (7.6161)
Adjust *R*-squared	0.4272	0.4396
Observations	1289	1289

^***, **, *^ represents significant at 1%, 5% and 10% levels respectively, with standard errors in brackets

## Discussion

This study provides valuable insights into the impact of social media on residents' preventive behaviours during the COVID-19 outbreak. Understanding the findings of this study could improve managers' knowledge about the factors that drive residents towards preventive behaviours, particularly during the COVID-19 outbreak. Online shopping was chosen as a specific preventive behaviour in this study for empirical evaluation.

### Theoretical implications

The literature has widely applied risk perception in the research on the formation mechanism of residents' online shopping, and the results indicate that risk perception is the main factor influencing residents' online shopping [20, 21]. However, risk perception is not the initial factor influencing residents' online shopping. This study applied and expanded upon Davis's TAM [16]. It examined how social media heterogeneity affects residents' preventive behaviour and included social media as the main means of disseminating risk information. The literature has rarely discussed this topic, particularly in the context of risk communication.

This study provides evidence of the importance of social media for influencing residents' preventive behaviour. Risk information is considered to be the initial factor influencing residents' behaviour and triggers residents' cognitive processes [2]. Social media is the main channel through which residents obtain risk information, especially for emerging diseases, as most residents lack direct experience [3]. There was a transient increase in residents' risk information needs during the COVID-19 outbreak, and residents used various information channels (i.e., self-media and official-media) to fulfil these unmet needs [51]. Residents had access to COVID-19 prevention strategies and increased awareness of the outbreak by acquiring risk information. This awareness lessens uncertainty in the face of the outbreak [52], and residents can combine the solutions available to the current situation and respond quickly to the COVID-19 outbreak.

There are significant differences in residents’ information perception due to the use of different information collection channels, which in turn affects their risk perception and choice of protective behaviour decisions [7, 8]. Aligning with and going beyond the existing studies, this study investigates the heterogeneous effects of different information sources in social media on residents' willingness and behaviour to shop online. Residents are more likely to accept risk information with high credibility. Previous studies have indicated that official-media is more credible than self-media [10]. Studies of U.S. adults sharing information related to novel coronavirus pneumonia have shown that people often share false statements about novel coronavirus pneumonia, partly because they do not adequately consider the accuracy of the risk information when sharing it [53]. Our findings show that the promotion effect of risk information provided by official-media is greater for residents' online shopping willingness and online shopping behaviour than that provided by self-media. Our results also suggest that official-media and self-media can collaboratively promote residents' online shopping willingness and online shopping behaviour. Self-media can compensate for official-media's slow dissemination, whereas official-media can compensate for self-media's lack of credibility.

This paper also explored the link between residents' preventive behaviour willingness and actual preventive behaviour. The linkage between intention and usage is frequently overlooked in TAM-based research studies since they tend to adopt intentions rather than actual use as outcome variables [22]. These findings show that there is a deviation between residents' online shopping willingness and online shopping behaviour. This study focused on two perceptions of the TAM (perceived usefulness, perceived ease-of-use), as these perceptions have been found to exert direct influence on residents' preventive behaviour along with willingness (not indirectly influencing behaviour via willingness) and have been found to influence how social media provides ease-of-use information and usefulness information about preventive behaviour, promoting the transformation of preventive behaviour willingness to actual preventive behaviour.

### Managerial implications

First, this study examined the heterogeneous effects of different information sources on residents' willingness towards preventive behaviour on social media. The findings indicate that both official and self-media positively promote residents' online shopping willingness and online shopping behaviour, with official-media having a stronger promotional effect than self-media. To encourage residents to actively adopt preventive behaviours, public health departments, governments and other institutions should attach importance to social media during times of crisis. Self-media disseminated risk messages with ambiguous rhetoric and reporting at the early stage of the epidemic, which influenced the residents' correct perception of risk facts [54]. Therefore, the government should especially control the credibility of risk information from social media to avoid incorrect risk information, inciting residents to adopt high-risk behaviours. Our results also suggest that official-media and self-media can collaboratively promote residents' online shopping willingness and online shopping behaviour. Self-media prioritized the release of risk information about COVID-19 to enhance residents' awareness of the current situation and compensate for the lag in risk information in the official-media. Governments and other institutions should also be encouraged to cooperate with doctors, specialists and opinion leaders to ensure the prompt provision of correct, relevant, crisis-related information to the public [55], which would help prevent the spread of rumours and other misinformation [56].

Second, there is a gap between residents' online shopping willingness and online shopping behaviour, which is supported by the findings of our study. Therefore, it is also important to guide the transformation of residents’ willingness to engage in protective behaviour to actual protective behaviour. Our findings showed that ease-of-use and usefulness of information significantly promoted the transformation of residents' online shopping willingness and online shopping behaviour. Public health departments, governments and other institutions should increase communication about the risk-reducing effects of preventive behaviours in times of crisis, which could enhance residents' perceptions of usefulness. Moreover, residents with prevention experience could be encouraged to provide guidance through offline guidance and video guidance, which could enhance residents' perceptions of ease-of-use.

## Conclusion

These results showed that residents actively adopted preventive behaviour during the COVID-19 emergency. Moreover, there is deviation between residents' willingness to engage in preventive behaviour and actual preventive behaviour. The results show that both official-media and self-media positively promote residents' online shopping willingness and online shopping behaviour, and the promotion effect of official-media is greater than the promotion effect of self-media. In addition, official-media and self-media can collaboratively promote residents' online shopping willingness and online shopping behaviour. The ease-of-use and usefulness of information significantly promoted the transformation of residents' online shopping willingness and online shopping behaviour.

### Supplementary Information

**Supplementary Material 1.**

## Data Availability

The datasets used and analyzed during the current study are available from the corresponding author on reasonable request.
