# Supplementary material for: Social media heterogeneity and preventive behaviours during the COVID-19 outbreak: a survey on online shopping
Source: BMC Public Health. 2024 Apr 29;24:1193. doi: 10.1186/s12889-024-18253-y (PMC11057092; doi:10.1186/s12889-024-18253-y)
Supplement: Supplementary file 1 — Supplementary Material 1. [file 12889_2024_18253_MOESM1_ESM.docx]

**Empirical Models**

This paper focuses on examining the heterogeneous effects of different information sources on residents' online shopping willingness and online shopping behaviour. Considering that the results of ordinary least squares (OLS) estimation are more understandable and easier to interpret, the OLS estimation is used in this paper. The model can be given as:

Model 1: $OSW_{i}=\alpha_{0}+\alpha_{1}\text{O}M_{i}+\alpha_{2}\text{S}M_{i}+\sum\alpha_{j+2}Control_{j,i}+u_{i}$ (1)

Model 2: $OSW_{i}=\lambda_{0}+\lambda_{1}\text{O}M_{i}+\lambda_{2}\text{S}M_{i}+\lambda_{3}\text{OM}_{i}\times S\text{M}_{i}+\sum\lambda_{j+3}Control_{j,i}+u'_{\text{i}}$ (2)

Model 3: $OSB_{i}=\partial_{0}+\partial_{1}\text{O}M_{i}+\partial_{2}\text{S}M_{i}+\sum\partial_{j+2}Control_{j,i}+u''_{i}$ (3)

Model 4: $OSB_{i}=\beta_{0}+\beta_{1}\text{O}M_{i}+\beta_{2}\text{S}M_{i}+\beta_{3}\text{OM}_{i}\times S\text{M}_{i}+\sum\beta_{j+3}Control_{j,i}+u'''_{i}$ (4)

Where *OM_i_* and *SM_i_* respectively represent official-media and self-media, *OSW_i_* and *OSB_i_* respectively represent the online shopping willingness and online shopping behaviour. *Control_i_* is a vector of control variables that may affect the online shopping willingness and online shopping behaviour. *OM_i_* × *SM_i_* represent the interaction term between official-media and self-media. *λ_3_ and β_3_* respectively reflect the effects of the interaction term between official-media and self-media on residents’ online shopping willingness and online shopping behaviour. $u_{i}$, $u'_{i}$, $u''_{\text{i}}$ and $u'''_{i}$are error terms.

In order to test the heterogeneous impact of different information content on social media on the transformation of residents' online shopping willingness and online shopping behaviour, the model can be expressed as:

Model 5: $OSB_{i}=\omega_{0}+\omega_{1}OSW_{i}+\omega_{2}EUI_{i}+\omega_{3}UI_{i}+\sum\omega_{j+3}Control_{j,i}+\theta_{i}$ (5)

Model 6: $OSB_{i}=\eta_{0}+\eta_{1}OSW_{i}+\eta_{2}EUI_{i}+\eta_{3}UI_{i}+\eta_{4}OSW_{i}\times EUI_{i}+\eta_{5}OSW_{i}\times UI_{i}+\sum\eta_{j+5}Control_{j,i}+\theta'_{i}$ (6)

Where *UI_i_* and *EUI_i_* respectively represent usefulness information and ease-of-use information. *OSW_i_* × *EUI_i_* represent the interaction term between online shopping behaviour and ease-of-use information. *OSW_i_* × *UI_i_* represent the interaction term between online shopping behaviour and usefulness information. *η_4_* and *η_5_* respectively reflect the moderating effect of ease-of-use information and usefulness information on the transformation of online shopping willingness to online shopping behaviour. $\theta_{i}$ and $\theta_{i}^{'}$ are error terms.
